# Human *Streptococcus suis* Outbreak, Sichuan, China

**DOI:** 10.3201/eid1206.051194

**Published:** 2006-06

**Authors:** Hongjie Yu, Huaiqi Jing, Zhihai Chen, Han Zheng, Xiaoping Zhu, Hua Wang, Shiwen Wang, Lunguang Liu, Rongqiang Zu, Longze Luo, Nijuan Xiang, Honglu Liu, Xuecheng Liu, Yuelong Shu, Shui Shan Lee, Shuk Kwan Chuang, Yu Wang, Jianguo Xu, Weizhong Yang, the Streptococcus suis study groups

**Affiliations:** * Chinese Center for Disease Control and Prevention, Beijing, People's Republic of China;; † State Key Laboratory for Infectious Disease Prevention and Control (ICDC), Beijing, China;; ‡ Beijing Ditan Hospital, Beijing, People's Republic of China;; § Sichuan Center for Disease Control and Prevention, Sichuan, People's Republic of China;; ¶ Jiangsu Center for Disease Control and Prevention, Nanjing, People's Republic of China;; # The Chinese University of Hong Kong, Hong Kong Special Administrative Region, People's Republic of China;; ** Centre for Health Protection, Hong Kong Special Administrative Region, People's Republic of China

**Keywords:** Keywords: Streptococcus suis, streptococcal toxic shock syndrome, outbreak, zoonoses, research

## Abstract

From mid-July to the end of August 2005, a total of 215 cases of human *Streptococcus suis* infections, 66 of which were laboratory confirmed, were reported in Sichuan, China. All infections occurred in backyard farmers who were directly exposed to infection during the slaughtering process of pigs that had died of unknown causes or been killed for food because they were ill. Sixty-one (28%) of the farmers had streptococcal toxic shock syndrome; 38 (62%) of them died. The other illnesses reported were sepsis (24%) and meningitis (48%) or both. All isolates tested positive for genes for *tuf*, species-specific 16S rRNA, *cps2J*, *mrp*, *ef*, and *sly*. A single strain of *S. suis* caused the outbreak, as shown by the identification of a single ribotype. The high death ratio was of concern; prohibiting backyard slaughtering ended the outbreak.

## Abstract 2

*Streptococcus suis* outbreak was associated with exposure to sick or dead pigs.

*Streptococcus suis* is a zoonotic microbe that can exist in pigs without causing illness but can occasionally cause disease. Serotype 2 is a dominant pathogenic serotype (*1*). Types 2 and 5 have been isolated from purulent lesions in the lungs and other extramammary sites in cattle, sheep, and goats (*2*). Infection may cause death in weaning piglets as well as growing pigs (*3*). The bacterium is isolated from an increasingly wide range of mammalian species, including horses, dogs, cats, and birds (*4*).

Sporadic cases of *S. suis* infection may occur in humans, the most common clinical manifestations included purulent meningitis, septicemia, arthritis, and endocarditis; some infections lead to sequelae such as deafness and ataxia (*5**–**7*). To date, ≈200 human cases have been reported in areas of intensive pig rearing (the Netherlands, Denmark) or areas where large quantities of pork are eaten (Hong Kong, Thailand, Vietnam) (*8*). Most reported cases of human *S. suis* infections were associated with contact with pigs or pork products (*5*). Human *S. suis* infections do not normally cause major outbreaks.

On July 11, 2005, a local hospital in Ziyang Prefecture of Sichuan Province reported a suspected case of hemorrhagic fever with renal syndrome. The patient was a 46-year-old male farmer with acute onset of high fever, lethargy, vomiting, and generalized purpura. The day before illness onset, the farmer slaughtered a pig that had died of an unknown cause. The farmer rapidly lapsed into coma. On further investigation, we identified 4 other patients with similar circumstances in the same hospital and more patients from other hospitals in the area. *S. suis* was isolated from blood cultures in some of these cases. We began an investigation of this outbreak to describe its epidemiologic, clinical, and microbiologic characteristics (*9*).

## Methods

### Epidemiologic Investigation

We reviewed medical records in all health care facilities in Ziyang Prefecture for patients admitted since June 10, 2005 (2 weeks before the onset of the first known case), with a diagnosis of septic shock or meningitis. On July 19, enhanced surveillance was introduced to include healthcare facilities in Ziyang and the 5 surrounding prefectures. We ordered all healthcare facilities to immediately report all new patients with clinical sepsis, meningitis, arthritis, or endocarditis and fever >37.3°C, who had epidemiologic risk factors (contact with sick pigs or any part, such as meat, skin, organs, or tissue, of pigs that had died of undetermined causes). Beginning on July 21, we made public announcements to encourage reporting. Public health workers interviewed patients, or surrogates for deceased patients, by using a questionnaire to collect demographic, clinical, and exposure information. Specimens (blood, cerebrospinal fluid [CSF], or postmortem tissue) were collected for laboratory investigation. We reviewed medical records to obtain supplementary clinical information. We placed all close contacts of case-patients, including family members and attending healthcare workers, under medical surveillance. We extended these surveillance procedures to all of Sichuan Province on July 25.

A probable case of *S. suis* infection was a compatible clinical illness (sepsis, meningitis, arthritis, or endocarditis), without laboratory evidence of infection by another organism, with history of contact with sick or dead domestic livestock (pigs, goats, or sheep) or another case-patient within 7 days before onset of symptoms. A confirmed case was defined as a compatible clinical illness regardless of exposure and the verification of *S. suis* isolated from a normally sterile site. We stopped the enhanced surveillance on August 18, two weeks after onset of the last case.

To assess the extent of underreporting, notification statistics on meningococcal meningitis were reviewed. Meningococcal meningitis is a statutorily notifiable disease in China. Because laboratory diagnosis was not routinely performed for all meningitis patients, the figures on suspected meningococcal cases could reflect nonmeningococcal meningitis caused by other bacteria, including *S. suis*. In this investigation, statistics in the 12 affected prefectures of Sichuan Province from January 2003 to August 2005 were reviewed.

### Laboratory Investigation

Specimens of blood, CSF, or postmortem tissue from human patients and blood or postmortem tissue from affected pigs were injected onto sheep blood agar and infusion broth (REF 237500, Oxoid, Basingstoke, UK) in 0.5% CO_2_ at 37°C. *S. suis* strains were identified by examining growth colony shape, followed by biochemical reactions with Vitek2 compact and API 20 strep (bioMérieux, Inc., Beijing, China) according to the manufacturer's instructions.

Polymerase chain reaction (PCR) was used to characterize selected genes of *S. suis* serotype 2. PCR products were purified by using the QIAquick PCR purification kit (Qiagen, Valencia, CA, USA), according to the manufacturer's protocol, and sequenced with an ABI Prism 3700 DNA instrument (Applied BioSystems, Foster City, CA, USA). The following genes were sequenced, in accordance with methods published elsewhere: 1) genus-specific gene segments, *tuf* sequence, and 2) the species-specific gene coding for 16S rRNA of *S. suis*, the gene coding for the capsule of *S. suis* serotypes 2 (*cps2J*) and 1/2, the muramidase-released protein gene (*mrp*), suilysin (*sly*), and the extracellular factor gene (*ef*) (*10**–**12*). Sequence data were analyzed by using a basic local alignment search tool (BLAST) search performed against sequences published by the National Center for Biotechnology Information (Bethesda, MD, USA).

Automated *Pvu*II and *Pst*I ribotyping was performed by using the RiboPrinter microbial characterization system (DuPont China, Shenzhen, China), with bacterial isolates grown overnight on brain-heart infusion blood agar. Template preparation, restriction enzyme digestion, gel electrophoresis, and Southern hybridization with an *Escherichia coli* rrnB rRNA operon probe were carried out with the RiboPrinter system. Images were developed with a charge-coupled-device camera and analyzed by using the RiboPrinter's customized software.

### Statistical Analysis

Percentage, proportion, and case-fatality ratios were calculated. The χ^2^ and rank sum tests were used to compare the case-fatality ratios and the median duration from exposure to onset between 2 groups, respectively, by using Stata version 8 (StataCorp LP, College Station, TX, USA). All probabilities were 2-tailed, and p<0.05 indicated significance.

## Results

The first case-patient was a 52-year-old male farmer from Yanjiang District of Ziyang Prefecture. On June 24, 2005, three days after he slaughtered and dressed a goat that had died of an unknown cause, fever (38.2°C), chills, abdominal pain, vomiting, generalized aching, and generalized purpura developed. Ten hours later, he died on the way to the hospital. The second case-patient was his neighbor who had similar symptoms on June 26 and died shortly after hospital admission. He had helped slaughter the goat with the first case-patient. Blood, tissue, and other specimens for culture were not available from these patients.

### The Outbreak

**Figure 1:**
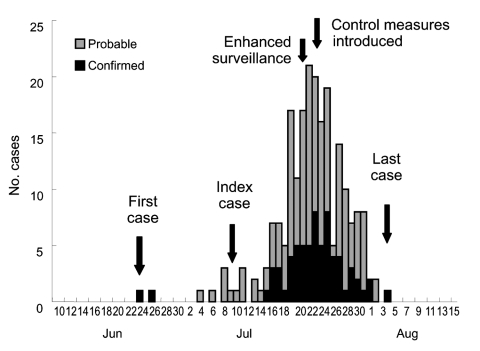
Epidemic curve showing the dates of onset for 215 human cases of *Streptococcus suis* infection, Sichuan, China (as of August 18, 2005).

**Figure 2:**
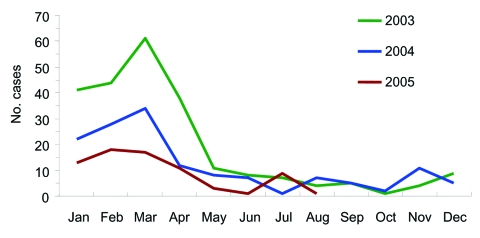
Suspected cases of meningococcal meningitis in the 12 affected prefectures in Sichuan.

Other cases of *S. suis* infection occurred in early July. The number of cases gradually increased, peaked during the second half of July, and dwindled rapidly thereafter (Figure 1). The decline coincided with new measures that prohibited domestic slaughter of sick pigs or pigs that died of any illness. These measures were implemented through provincial legislation and enforced with prosecution. The last case-patient had onset of illness on August 4. By August 18, two weeks after the date of onset of the last case, we had identified 215 cases (66 laboratory confirmed, 149 probable); 39 persons died in Sichuan Province. This finding compares to an expected number of suspected meningococcal disease cases of 10 per month reported through routine surveillance during the summer months (June to August) from 2003 to 2005 (Figure 2).

Cases of *S. suis* infection predominantly involved adult male farmers with recent exposure to sick pigs or carcasses of pigs that had died of unknown illnesses (Table 1). The most common methods of exposures were slaughtering a sick pig or preparing a pig carcass for meat, hides, and other pig products. Forty-eight percent of case-patients had wounds on their hands at the time of slaughter or carcass preparation. Illnesses that fit the case definition did not develop in close contacts of case-patients who did not participate in the slaughter, carcass preparation, or carcass disposal. None of the 417 healthcare workers who cared for case-patients became infected, and no similar clinical illness developed.

**Table 1:** Demographic features and exposure history of 215 *Streptococcus suis* patients in Sichuan, China, 2005

Characteristic		Probable cases, n = 149	Confirmed cases, n = 66	Total, N = 215
Demographic features				
	Male (%)	120 (81)	60 (91)	180 (84)
	Median age, y (range)	54 (26–82)	57 (33–81)	54 (26–82)
	Farmer (%)	145 (97)	62 (94)	207 (96)
	Other occupation (%)*	4 (3)	4 (6)	8 (4)
	Wounds on hands during pig exposure (%)	74 (50)	30 (45)	104 (48)
Exposure history 7 days before onset of symptom, no. (%)				
	Slaughtered sick pig or goat	93 (62)	47 (71)	140 (65)
	Prepared carcasses of pig or goat without slaughtering	44 (30)	16 (24)	60 (28)
	Other exposure to sick pigs	12 (8)	3 (5)	15 (7)
	Only ate meat from sick/dead pig/goat	0	0	0
	Only contact with known case-patients	0	0	0

*Fed sick pigs or handled carcasses (e.g., sold carcasses, buried carcasses) of pigs that died from unexplained illness.

**Figure 3:**
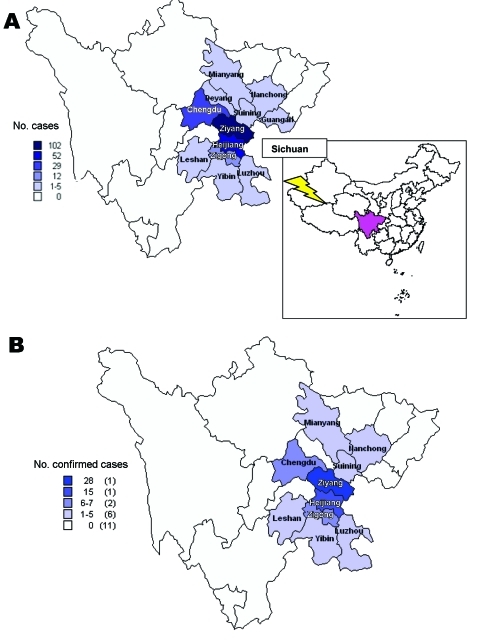
Geographic distribution of *Streptococcus suis* cases in Sichuan Province, China, relating to (A) all reports, and (B) 66 laboratory-confirmed cases alone (as of August 18, 2005).

The residences of *S. suis* case-patients were widely distributed in 203 villages of 12 prefectures in Sichuan Province. In 194 villages, only 1 case was identified (Figure 3). In Ziyang, Yibin, and Chengdu, 2 case-patients in 1 village each were identified. The Ziyang cluster was composed of the first 2 case-patients in the outbreak. The Yibin cluster consisted of 1 farmer who slaughtered a sick pig and became ill with streptococcal toxic shock syndrome (STSS); another person who processed meat from the same pig became ill with meningitis and recovered. In Chengdu, 2 persons slaughtered a pig that had died of an unexplained illness; *S. suis* infection developed in both persons and they recovered. In 6 additional villages, >2 cases of *S. suis* infection occurred in patients who did not know each other and had no common exposure to a pig.

### Clinical Patterns

All 215 affected persons were previously healthy adults. We observed 3 distinct clinical symptom settings (Table 2). First, 28% of patients had sepsis characterized by acute onset of fever, chills, headaches, dizziness, malaise, abdominal pain, and diarrhea. In some severe cases, patients became comatose. Second, 48% of patients had meningitis characterized by headache, stiff neck, and other signs of acute meningitis. Some meningitis patients also had disseminated intravascular coagulation and coma. Third, 28% of patients had STSS that has been described in other forms of streptococcal infections (*13*) and met the criteria established by Centers for Disease Control and Prevention (*14*). Patients with STSS had a 62% case-fatality ratio compared to 0.6% for other clinical forms of streptococcal infections. Fatal STSS cases progressed from onset to death in a median of 25 hours (range 8 hours to 10.5 days). Discharge records available on 85 survivors in all 3 clinical symptom settings showed a median interval from onset to recovery of 15 days (range 5–36 days).

**Table 2:** Clinical symptoms and case-fatality ratio of 215 *Streptococcus suis* patients in Sichuan Province, China, 2005*

Clinical symptom	Probable		Confirmed		Total	
	No. (%)	No. deaths (case-fatality %)	No. (%)	No. deaths (case-fatality %)	No. (%)	No. deaths (case-fatality %)
Sepsis	45 (30)	0	7 (11)	0	52 (24)	0
Meningitis	69 (46)	1 (1)	33 (50)	0	102 (48)	1 (1)
STSS	35 (24)	23 (66)	26 (39)	15 (58)	61 (28)	38 (62)
Total	149 (100)	24 (16)	66 (100)	15 (23)	215 (100)	39 (18)

*Streptococcal toxic shock syndrome (STSS) is defined according to the 1996 criteria established by the Centers for Disease Control and Prevention, Atlanta, GA, USA, which include hypotension (systolic blood pressure <90 mm Hg for adults) and multiorgan involvement characterized by >2 of the following: renal impairment, coagulopathy, liver involvement, acute respiratory distress syndrome, generalized erythematous macular rash that may desquamate, soft-tissue necrosis, including necrotizing fasciitis or myositis, or gangrene. Difference in case-fatality ratio between STSS and other clinical symptoms p<0.001 by χ^2^ test.

**Figure 4:**
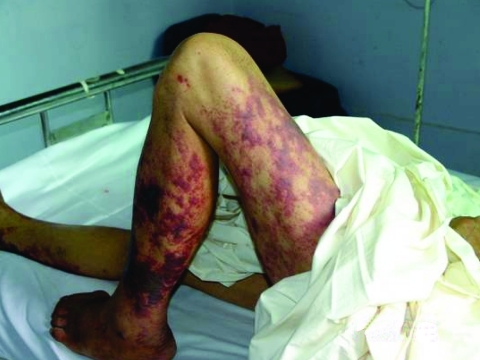
Photograph of a *Streptococcus suis* patient's legs with streptococcal toxic shock syndrome, featuring purpura and evidence of gangrenous changes in the calf extending down to the foot.

We determined exposure and onset times for 63 of the 66 laboratory-confirmed cases. Of the 215 cases, we could determine exposure time and onset times for 203. The median interval between exposure and onset was 2.2 days (range 3 hours to 14 days). The patients with STSS had a shorter incubation period and a higher frequency of gastrointestinal symptoms, coma, and petechiae and ecchymoses than did other patients (Table 3). Eight percent of non-STSS cases had petechiae or ecchymoses, and 9% had hypotension but did not fully fit the criteria for STSS. Typical skin manifestations are shown in Figure 4. Leukocytosis and thrombocytopenia were found in more than half of the patients (Table 4). Liver impairment was found in 74%, and renal function impairment was found in 19% of the patients who had been tested. CSF abnormalities compatible with purulent meningitis were found in 31 (40%) of 77 patients who had a lumbar puncture.

**Table 3:** Clinical symptoms of human cases of *Streptococcus suis* infection in Sichuan, China, 2005*

Symptoms and signs	STSS, no. (%) (n = 61)	Non-STSS, no. (%) n = 154)	Total, no. (%) (N = 215)
Fever (temperature >37.3°C)	61 (100)	154 (100)	215 (100)
Chills	48 (79)	128 (83)	176 (82)
Headache	38 (62)	110 (71)	148 (69)
Myalgia	30 (49)	73 (47)	103 (48)
Vomiting	41 (67)	80 (52)	121 (56)
Abdominal pain	24 (39)	33 (21)	57 (27)
Diarrhea	28 (46)	22 (14)	50 (23)
Coma	16 (26)	26 (17)	42 (20)
Petechiae, ecchymosis†	37 (61)	12 (8)	49 (23)
Neck rigidity	4 (7)	50 (32)	54 (25)
Kernig positive	1 (2)	27 (18)	28 (13)
Brudzinski positive	2 (3)	17 (11)	19 (9)
Hypotension (blood pressure <90 mm Hg)†‡	25 (93)	2 (9)	27 (55)

*STSS, streptococcal toxic shock syndrome. Median duration between exposure and onset (range) was 1.6 d (9 h–9 d) for STSS, 2.5 d (6 h–14 d) for non-STSS, and 2.2 d (3 h–14 d) for total sample. Difference between median incubation periods of STSS and non-STSS, p<0.05, rank sum test. †Difference in frequency between STSS and non-STSS, p<0.001, χ^2^. ‡Not conducted in every patient.

**Table 4:** Initial laboratory test results of human cases of *Streptococcus suis* infection in Sichuan, China, 2005*

Result	STSS (n = 61)	Non-STSS (n = 154)	Total (N = 215)
Leukocytosis, >10 × 10^9^/L, no. (%)†	26 (52)	97 (71)	123 (66)
Mean leukocyte count (range)	12.3 (1.0–47.8)	14.5 (2.0–64.0)	13.9 (1.0–64.0)
Thrombocytopenia, <100 × 10^6^/L, no. (%)†	34 (74)	53 (45)	87 (53)
Mean platelet count (range)	94.7 (10.0–287.0)	118.5 (13.3–689.0)	115.0 (4.4–689.0)
Liver function impairment, no. (%)†‡	27 (90)	50 (68)	77 (74)
Renal impairment, no. (%)†§	20 (59)	3 (3)	23 (19)
CSF abnormality, no. (%)†¶	0	31 (46)	31 (40)

*STSS, streptococcal toxic shock syndrome. †Not all tests were conducted on all patients. ‡Alanine aminotransferase >2× upper limit of normal. §Creatinine >177 μmol/L for adults or >2× upper limit of normal for age. ¶CSF, cerebrospinal fluid, protein >0.45 g/L, glucose <2.5 mmol/L, and >8 leukocytes × 10^6^/L.

Postmortem examination of 4 STSS patients (2 confirmed and 2 probable infections) showed features of disseminated intravascular coagulation. Evidence of multiple organ damage was observed, primarily involving kidneys, adrenal glands, lungs, liver, pancreas, and heart. Histologic findings included microthrombosis (hyaline thrombus) in organ capillaries; necrosis of parenchymal cells; and congestion, exudate, and hemorrhage of interstitial vessels of kidneys, lungs, and other organs.

### Microbiologic Investigations

We collected 348 specimens of blood (271), CSF (53), and tissue (24) from postmortem examination of 172 case-patients. We isolated *S. suis* from blood (36), CSF (27), and postmortem liver, spleen, and heart tissues (3). Fifty-five (83%) of the confirmed patients were diagnosed after July 23, 2005, representing 42% (55/130) (data not shown) of all tested samples, compared to that of 26% (11/42) (data not shown) before enhancement of surveillance.

**Figure 5:**
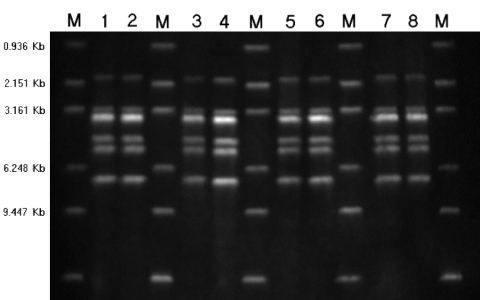
Ribotyping of *Streptococcus suis* serotype 2 isolates by *Pvu*II restriction. Lane 1, deceased pig isolate SC5; lane 2, deceased pig isolate SC16; lane 3, patient isolate SC154; lane 4, patient isolate SC160; lane 5, patient isolate SC175; lane 6, patient isolate SC179; lane 7, patient isolate SC204; lane 8, patient isolate SC206; M, molecular size standard.

Isolates from 66 patients and 3 diseased pigs featured pure growth of tiny α-hemolytic colonies on sheep blood agar. The colony shape and biochemical reactions by Vitek2 compact and API-Strep were all compatible with that of *S. suis*. In the investigations after July 23, 2005, the results matched the suggested key indicators for the pathogen, including Voges-Proskauer negativity, hydrolysis of esculin, trehalose positivity, negativity for growth in 6.5% NaCl, and absence of β-hemolysis on sheep blood agar. PCR on all isolates showed gene coding for *tuf*, species-specific 16S rRNA of *S. suis*, genes coding for the capsule of *S. suis* serotypes 2 (*cps2J*) and 1/2, and *mrp*, *sly*, and *ef*. The PCR products of virulence genes of all 69 strains were sequenced. These were identical to the sequences published by the National Center for Biotechnology Information. On further investigation, only a single ribotype for either *Pvu*II or *Pst*I restriction was identified among the 25 *S. suis* type 2 isolates, including 22 from patients and 3 from diseased pigs (Figure 5), indicating a single clonal strain as the source of the infection (*1*).

## Discussion

We report an unprecedented outbreak of human *S. suis* serotype 2 infection involving 215 patients in Sichuan Province during the summer of 2005. This outbreak was characterized by the large number of patients involved, distinctive clinical manifestations, and the major challenges facing public health authorities in both surveillance and control.

This is the largest recorded outbreak of *S. suis* infection in humans. From late July to early August 1998, an outbreak of 25 cases with 14 deaths occurred in Jiangsu Province, China, from serotype 2 infection (*15*). The size of the outbreak in our study was probably related to a local farming practice. In Sichuan, a sizable swine population was found in small backyard farms; each family kept only a few animals. Farmers habitually slaughtered sick pigs for human consumption. Beginning mid-June 2005, a major outbreak of *S. suis* killed 647 pigs in almost the same areas as the outbreak in humans (*16*). The outbreak in swine peaked around July 20 with ≈4 dead pigs in each affected village. *S. suis* caused 98% of the deaths of these pigs in Sichuan during this period (*17*). All infected pigs came from backyard farms, usually with 1 sick pig in the herd. A pathogenic strain could have spread by distributing infected piglets to the backyard pig farms and then propagated among healthy pigs. The single ribotype identified in the investigation lends support to this theory.

The main risk factor for *S. suis* infection in the outbreak was direct involvement in slaughtering sick pigs and preparing carcasses of pigs that died of unknown causes. Unlike professionals in modern abattoirs, the local farmers did not wear protective gear or gloves. Normally 1–2 persons carried out the procedure, which involved bloodletting though a neck artery, manually inflating the carcasses, scalding the pigskin with ≈80°C water, and splitting and shaving the skin with large knives. Scalding and shaving were often performed together. The farmers then sliced the meat into smaller pieces before cooking for food. The complete process of slaughtering could take >1 hour. Our study demonstrated that all patients had been infected during direct contact with blood or tissues of sick or dead pigs. Often this may have occurred through direct exposure of skin wounds. Droplet exposure may also have occurred during slaughter or processing of carcasses, but we could not document this occurrence. The observed risk factors were consistent with those reported in other studies (*5**,**6*). No evidence of infection from eating cooked pork from these pigs was observed. The uncooked meat was shared with neighboring families, but these villagers normally do not eat raw meat or raw animal viscera. Person-to-person transmission was highly unlikely since we found no disease in family members, neighbors, or healthcare workers who had not been exposed to sick or dead pigs.

One missing link in the outbreak is the exact relationship between the dead goat in the early cluster of patients and the subsequent propagation of the *S. suis* infections. We could not confirm these 2 cases microbiologically. We speculate that *S. suis* caused these 2 early human infections because they occurred shortly after exposure to the dead goat, and because clinical manifestations in the 2 patients were similar to those of others in the outbreak. Human *S. suis* infection after exposure to sick goats has not been reported, despite isolation of the organism from these animals (*2*). In backyard farms where different animals are kept together, *S. suis* infection could have been transmitted between pigs and goats. Animal surveillance would help establish the role of animals other than pigs in carriage of the bacteria and the potential for causing human infections.

Clinically, 3 distinctive forms of human *S. suis* infection occurred, namely, STSS, sepsis, and meningitis. STSS has not been reported from *S. suis* infection, although it has been previously described in other streptococcal infections (*13*) and *Staphylococcus aureus* infections (*18*). Unusual STSS-like illnesses brought the outbreak to the attention of local health authorities. A dose effect may explain the relatively high proportion of STSS in this outbreak. While other explanations like comorbid conditions, e.g., asplenia, diabetes mellitus, alcoholism, and malignancy (*19*), have been reported, this was not the case for the outbreak in our study, which involved previously healthy adults.

Laboratory examination confirmed virulence factors in the *S. suis* isolates from this outbreak. They include *mrp*, *sly*, and *ef*, although their precise clinical role has not yet been shown. These isolates (*mrp*+, *ef*+, *sly*+) are related to European strains that are considered to be more virulent than North American strains (*1*). Genome analysis could determine if novel virulence genes were involved.

Finally, the high number of deaths due to STSS is a cause for concern. Prompt institution of effective responses to human *S. suis* outbreaks is a public health challenge, especially in rural China. Timely diagnosis is difficult. In our study, only 31% of the cases were laboratory confirmed. Suboptimal access to health services, personal delay in seeking treatment, underutilization of blood cultures in local hospitals, and self-administration of antimicrobial drugs may explain the relatively low proportion of culture-positive human cases, especially during the early phase of the outbreak. Many patients died without having sought treatment from any health facility. As for the investigation process, the existing surveillance system that covers meningitis is not robust enough to alert public health personnel of the impending threat of *S. suis*. Control measures included prohibiting by law of slaughtering, eating, selling, and transporting deceased or sick pigs. Subsidies were offered to families to support hygienic handling of deceased or sick pigs and to patients for medical care. Village heads were held accountable for illegal slaughtering in their village. These measures were supplemented with disinfection of affected backyard farms. Public education campaigns were staged to increase awareness of how to prevent and control human *S. suis* infections. In the long run, the prevention and control of swine infection should form the more strategic component of the public health program. Surveillance systems should be established to alert farmers and the general public if an infection outbreak in pigs is recognized (*19*).
